# Single-cell transcriptome analysis reveals elongation and ossification characteristics of antlers

**DOI:** 10.3389/fvets.2025.1658210

**Published:** 2025-11-26

**Authors:** Ruobing Han, Qianghui Wang, Heping Li

**Affiliations:** 1 College of Wildlife and Protected Area, Northeast Forestry University, Harbin, China; 2 Key Laboratory of Forest Resources Conservation and Utilization in the Southwest Mountains of China, Ministry of Education, Key Laboratory for Conserving Wildlife with Small Populations in Yunnan, College of Forestry, Southwest Forestry University, Kunming, China

**Keywords:** single-cell, antler elongation, differentiation trajectory, ligand–receptor interaction pairs, antler ossification

## Abstract

**Introduction:**

Antlers are specialized bony appendages in mammals that exhibit the remarkable ability to periodically regenerate from the pedicles and then rapidly elongate to form bone. Previous studies have demonstrated that this intricate and precise biological process involves the coordination of multiple cell types, whose understanding is central to a wide range of research areas, particularly those investigating bone growth and mineralization. Recent studies have shed light on the cellular composition in the rapid elongation and regeneration of antlers. However, the differentiation trajectories of mesenchymal and cartilage tissues in the antler tip at the single-cell resolution remain to be elucidated.

**Methods:**

The mesenchymal and cartilage tissues of a healthy 5-year-old male sika deer (*Cervus nippon*) were collected for single-cell RNA sequencing.

**Results:**

We generated a single-cell profile of the antler tip containing 11 distinct cell types. A novel transitional cell exhibiting multiple cellular characteristics was identified. A total of three putative differentiation trajectories were identified for antler elongation and ossification, revealing that stem-cell-like state cells have the potential to differentiate into multiple cell types, including chondroblasts, chondrocytes, mural cells, and endothelial cells. We emphasize that the elongation and ossification of antlers are coordinated by various cells, among which some vital ligand–receptor interaction pairs were identified to be shared by the vast majority of cell types, such as CADM1/CADM1, TGFB1/TGFBR3, LGR4/RSPO3, and HLA-C/FAM3C ligand–receptor interaction pairs.

**Conclusion:**

Taken together, the present study provides insights into rapid antler elongation and ossification at single-cell resolution and deepens our understanding of the differentiation process of antlers.

## Introduction

Only antlers have the unique capacity to grow rapidly and regenerate periodically every year. Previous studies have demonstrated that antlers are constantly undergoing growth and ossification through the coordinated activities of velvet, mesenchymal, cartilage, transition, and pre-cartilage cells (1). In addition, the skin, nerves, blood vessels, and fibrous tissue of the antler also undergo rapid growth. The rapid elongation of the antler is primarily driven by the proliferation of mesenchymal cells and the hypertrophy of chondrocytes (1–5). This also enables antler growth rates in the majority of deer species to exceed 2 cm/d during the rapid growth period (6). Therefore, antlers are excellent organ models for researchers to study mysterious biological phenomena such as the molecular mechanisms of ossification, rapid growth, and periodic regeneration (7).

Antler generation is widely considered a stem cell-based process (8). Antler stem cells (ASCs) possess the potential for self-renewal and differentiation into multiple cell types, including mesenchymal cells, chondrocytes, and osteoblasts (9), which contribute to the growth and mineralization of antlers (10). In addition, as a special bony appendage, the rapid growth of the antler is an essential process of endochondral ossification. Previous histological examinations have identified many cell types involved in rapid antler elongation (11, 12), among which mesenchymal stem cells can differentiate into chondrocytes through *in vitro* cell culture and differentiation experiments (13). This process is accompanied by thinning of the mesenchymal tissue and thickening of the cartilage tissue. Finally, the antler is completely ossified into bone. Recently, an increasing number of cells have been identified during the rapid growth and regeneration of antlers, including proliferating cells, immune cells, and macrophages (14–16). So far, the cellular composition and dynamic gene expression during antler regeneration have become increasingly clear. Among them, chondrocytes primarily secrete cartilage matrix and play a crucial role in antler elongation, while osteoblasts secrete the bone matrix and can calcify it, facilitating antler hardening and completing the entire regeneration process (17, 18). However, little is known about the differentiation path between the mesenchymal tissue and the cartilage tissue of the antler tip, and the cellular composition and oscillatory gene expression involved in this process also need to be further elucidated.

Therefore, we performed an unbiased clustering and comprehensive analysis of the mesenchymal and cartilage tissues in the antler tips during the rapid growth period of sika deer antlers using single-cell RNA-sequencing (scRNA-seq). A total of 11 distinct cell types were identified in the antler tips, and putative differentiation trajectories of the antlers were constructed, including the differentiation processes from stem cell-like state cells to chondroblasts and chondrocytes, and from stem cell-like state cells to mural cells and endothelial cells, paving the way to further elaborate, how various cells synergistically regulate the ossification and rapid growth of antlers. This study also highlights the importance of the interdependence and collaborative roles of diverse cells during antler elongation and ossification, emphasizing their significance for bone growth research, developmental biology, and potential regenerative medicine applications.

## Methods

### Collection of antler tissues

To elucidate gene expression changes in specific cell types and explore the mechanisms of antler elongation and ossification at the molecular level, we collected samples from a healthy 5-year-old male sika deer (*Cervus nippon*) from a commercial deer farm in Harbin, China. Antler tip tissues were collected according to the routine velveting procedure during the rapid growth period of the antler (Supplementary Figure S1). We then isolated the mesenchymal and cartilage tissues, cut them into small pieces, and stored them in liquid nitrogen for further use.

### Single-cell library construction and sequencing

Single nuclei were isolated from the mesenchymal and cartilage tissues using Nuclei EZ Lysis Buffer, supplemented with the protease inhibitor and RNase inhibitor. The single-nuclei suspensions were loaded onto a 10x Chromium chip according to the manufacturer’s instructions for the 10X Genomics Chromium Single-Cell 3′ kit (V3). Single-cell libraries were constructed based on the standard protocol and sequenced on an Illumina NovaSeq 6000, llumina, USA.

### Unbiased clustering analysis

We used the Cell Ranger pipeline (V6.1.1) to perform sample demultiplexing, barcode processing, and single-cell 3′ gene counting. The scRNA-seq data obtained were aligned to a high-quality reference genome of the male sika deer (19), and Seurat (version 4.1.0) (20) was used for dimensional reduction, clustering, and analysis of the scRNA-seq data. The expression levels were calculated through normalization. In addition, the quality control thresholds for the data were as follows: (1) genes expressed in at least three cells; (2) cells expressing between 500 and 5,000 genes; (3) cells with at least 500 UMI counts; and (4) cells where the percentage of mitochondrial DNA-derived gene expression was below 25%. We then used DoubletFinder (21) to remove doublets using the default parameters. We identified marker genes using the FindAllMarkers function in Seurat. Uniform Manifold Approximation and Projection (UMAP) was used to visualize the results of the single-cell transcriptome profile.

### Differential gene expression (DGE) and GO enrichment analysis

We used the FindAllMarkers function in Seurat to analyze DEGs among different cell types. Genes with |log_2_FC| ≥ 0.26 and an adjusted *p*-value of <0.05 were defined as Differentially Expressed Genes (DEGs). To investigate the function of DEGs across diverse cell types, GO enrichment analysis was performed. Specifically, we applied the clusterProfiler package (22) to construct the OrgDb of the sika deer and performed GO enrichment analysis using the enrichGO and enricher functions.

### Pseudotemporal analysis

We collected differentially expressed genes across different cell types and used the UMI count matrix of these different cell types [endothelial cells, antler blastema progenitor cells (ABPCs), mesenchymal cells, ASCs, transitional cells, chondroblasts, and chondrocytes] as the input file. We applied Monocle3 (23) to construct pseudotemporal trajectories of antler tips based on the tutorial. The scRNA data were normalized, and the batch effects were removed to ensure the accuracy and dependability of the subsequent analyses. Subsequently, the expression profiles of various cell types were reduced to two dimensions. We then identified the top marker genes in each cell. Finally, the pseudotemporal trajectory was obtained and visualized.

### Cell communication analysis

To evaluate ligand–receptor interaction relationships in the scRNA-seq data, we conducted a cell communication analysis using CellPhoneDB (24), which is a public database storing human ligand–receptor interactions. Briefly, we used Ensemble to identify homologous genes between sika deer and humans to analyze interaction relationships among diverse cells. The same cell types were aggregated, and ligand–receptor interaction pairs were predicted between two cell types based on the expression of the receptor in one cell type and the expression of the ligand in the other cell type. Notably, only ligand–receptor pairs with a cellular proportion of gene expression greater than 10% were included in the analysis. We then randomly arranged all cells to form a new cell population. The average value was obtained by calculating the average expression level of ligands in these cell types and the average expression level of receptors in the cell types that interact with them, with the process repeated multiple times. Cell–cell communication was predicted based on the number of significant ligand–receptor pairs enriched between two cell types. Finally, we visualized the interaction networks between different cell types via iTALK (25).

## Results

### The single-cell transcriptomic landscape of mesenchymal and cartilage tissues in the antler tip

**Figure 1:**
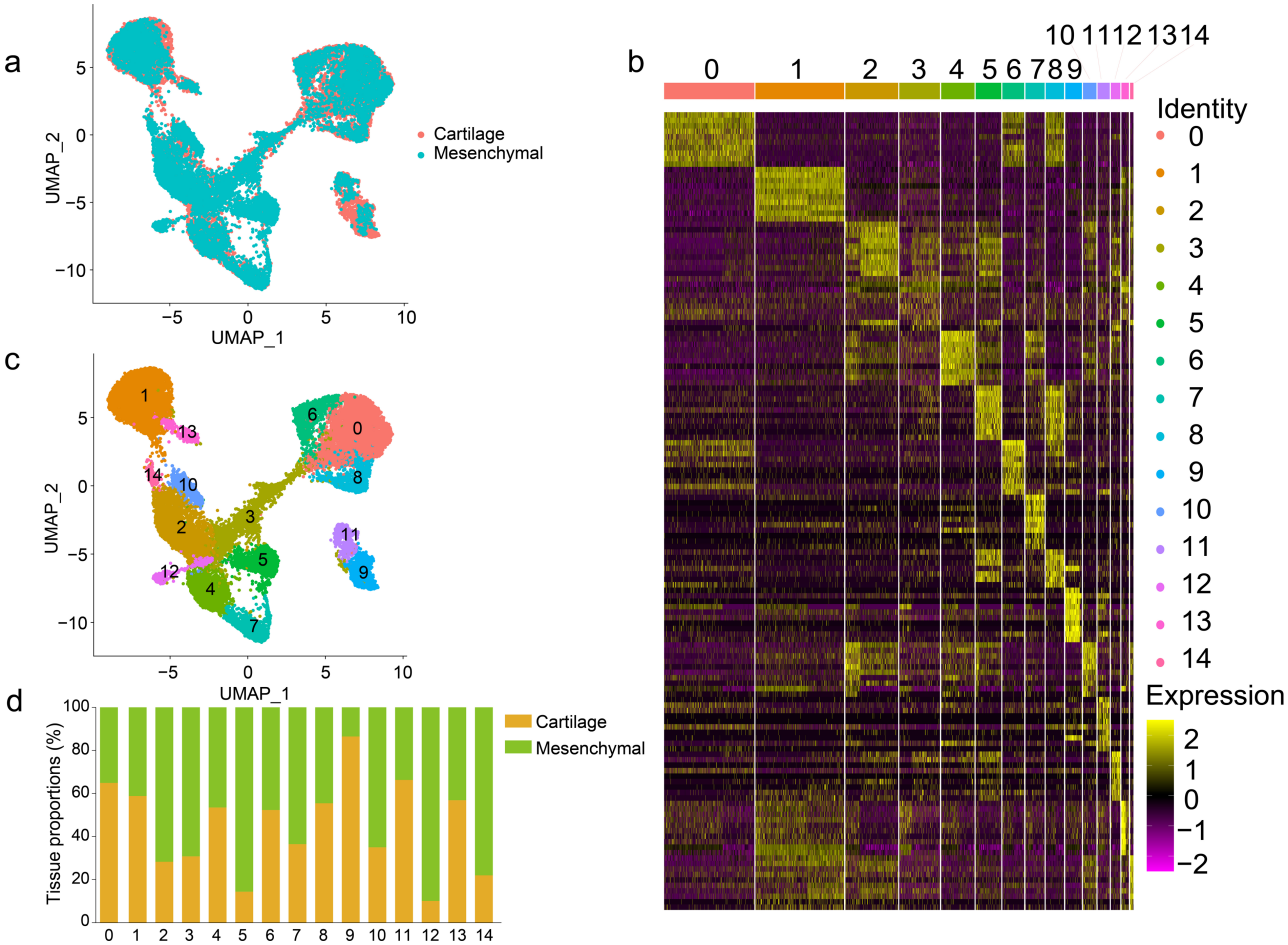
UMAP plot of the antler tip. **(a)** UMAP plot of the mesenchymal and cartilage tissues of the antler tip. Different colors indicate different samples. **(b)** Expression heatmap showing the highly expressed marker genes in the 15 clusters. **(c)** UMAP plot of the 15 clusters in the antler tip. Different clusters are marked by different colors. **(d)** Proportions of the 15 clusters in the mesenchymal and cartilage tissues of the antler tip.

To gain a comprehensive understanding of antler elongation and ossification, we performed scRNA-seq on two tissues that are critical for antler elongation and ossification, namely, mesenchymal and cartilage tissue of the antler tip. After cDNA amplification, library construction, and sequencing, a total of 11,663 cells were derived from the mesenchymal tissue, and 11,552 cells were acquired from the cartilage tissue. Rigorous filtering criteria were applied to the integrated dataset of 23,215 cells (Figure 1a). A high-quality single-cell transcriptional profile was generated from 20,266 filtered cells, with 3,145 mean reads per cell and 2,528 median genes per cell (Supplementary Figures S2a–c). We then detected 15 clusters from the single-cell datasets based on gene expression similarity, which reflected the relationships among these 15 clusters to some extent (Figures 1b,c). As expected, the same cell types from different tissues were often mixed, and the 15 clusters were shared between the mesenchymal and cartilage tissues (Figure 1d), indicating a certain degree of similarity in the cellular composition of the cartilage and mesenchymal tissues. In short, the transcriptome profile at single-cell resolution was helpful in elucidating the cellular composition of the antler tip and improved our understanding of antler elongation and ossification.

### Cell type annotation of the antler tip

**Figure 2:**
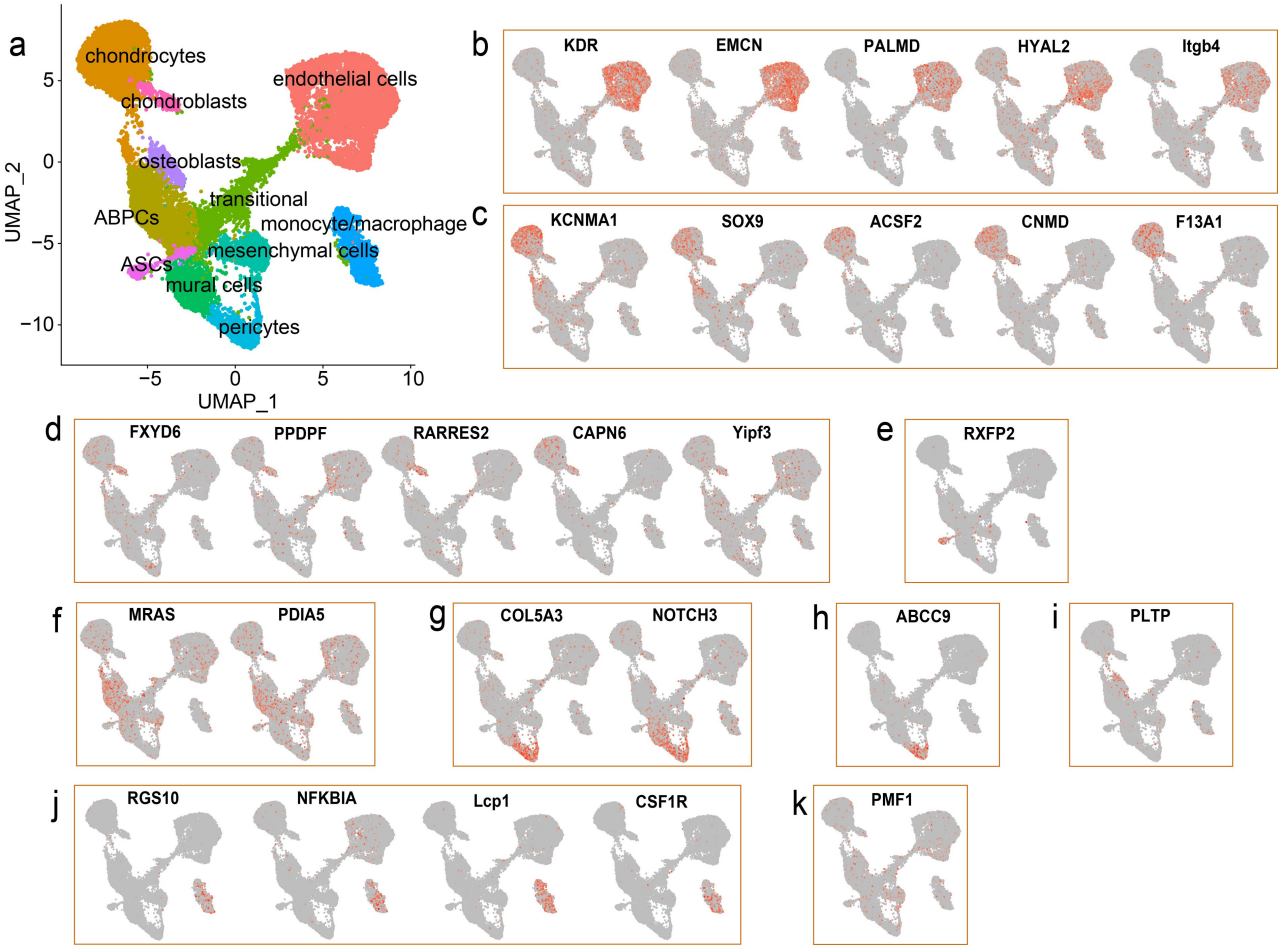
scRNA-seq facilitates identification of the main cell types in the antler tip. **(a)** UMAP visualization of the integrated scRNA-seq data from the mesenchymal and cartilage tissues. Each dot represents a single cell, and different cell types are marked by different colors. **(b)** UMAP visualization of the marker genes used to identify endothelial cells. **(c)** UMAP visualization of the marker genes used to identify chondrocytes. **(d)** UMAP visualization of the marker genes used to identify chondroblasts. **(e)** UMAP visualization of the marker genes used to identify ASCs. **(f)** UMAP visualization of the marker genes used to identify ABPCs. **(g)** UMAP visualization of the marker genes used to identify mural cells. **(h)** UMAP visualization of the marker genes used to identify pericytes. **(i)** UMAP visualization of the marker genes used to identify osteoblasts. **(j)** UMAP visualization of the marker genes used to identify monocytes/macrophages. **(k)** UMAP visualization of the marker genes used to identify mesenchymal cells.

To annotate the cell type of each cluster in the antler tip, marker genes for diverse cell types were collected from previous studies. A total of 11 distinct cell types were identified across the 15 clusters by combining these marker genes with CellMarker (Figure 2a) (26). We defined cluster 0, cluster 6, and cluster 8 as endothelial cells based on the high expression of the marker genes *KDR*, *PALMD*, and *EMCN* (15) (Figure 2b), and some integrin-encoding genes (*ITGA6*, *ITGB1*, and *ITGB4*) were also highly expressed in the endothelial cells. It is also noteworthy that the endothelial cells accounted for the highest proportion in both the mesenchymal and cartilage tissues of the antler tip (Supplementary Figure S3). We defined cluster 1 and cluster 14 as chondrocytes based on several marker genes, such as *ACSF2*, *CNMD*, *KCNMA1*, and *SOX9* (16) (Figure 2c). Among them, *SOX9* was also found to be highly expressed in cluster 13. As a member of the SRY family, *SOX9* plays a pivotal role in chondrogenesis, differentiation, and bone development (27–29). We then identified chondroblasts based on the expression of *PPDPF* and *RARRES2* (16) (Figure 2d). In addition, numerous genes closely related to bone development were identified in chondroblasts and chondrocytes, indicating the unique characteristics of chondroblasts and chondrocytes. Numerous members of the collagen family were also found to be highly expressed in chondroblasts and chondrocytes, such as *COL11A1*, *COL12A1*, *COL1A1*, *COL1A2*, *COL24A1*, *Col27a1*, *COL5A2*, *COL9A1*, *COL11A2*, *COL2A1*, *COL3A1*, *Col6a1*, and *COL9A2*. Cluster 12 was defined as antler stem cells (ASCs) based on the specific expression of the unambiguous marker gene relaxin family peptide receptor 2 (*RXFP2)* (Figure 2e), which has been associated with the horn phenotype (30, 31). Antler blastema progenitor cells (ABPCs) were also characterized based on the expression of *MRAS* and *PDIA5* (16) (Figure 2f). In addition, a large number of proliferative cells were identified in the single-cell transcriptomic landscape, such as pericytes and mural cells, which play vital roles in regulating the rapid growth and regeneration of the antler (15). Specifically, we annotated cluster 4 as mural cells due to the expression of the marker genes *NOTCH3* and *COL5A3* (15) (Figure 2g). Pericytes, a subpopulation of mural cells, were identified based on the specific expression of the marker gene *ABCC9* (15) (Figure 2h). In addition, osteoblasts were profiled in our transcriptomic landscape based on the expression of the marker genes *HHIP*, *PLTP*, *Col13a1*, and *FGFR1* (16) (Figure 2i). We then identified monocytes/macrophages based on the expression of *RGS10*, *NFKBIA*, and *CSF1R* (15) (Figure 2j), which may play a crucial role in regulating wound healing and tissue repair in antlers (32). We also annotated mesenchymal cells according to their exclusive expression of PMF1 (16) (Figure 2k). Finally, cluster 3 was found to have no obvious and distinct marker genes, and it displayed multiple marker genes of mesenchymal cells and ABPCs (top 10 marker genes: LUM, COL3A1, COL1A1, Rrbp1, MAP7D2, DMRTC2, RPL28, and COL5A1). Cluster 3 had marker genes of multiple cell types, including endothelial cells, chondroblasts, and chondrocytes, and was considered a transitional cell with mixed marker genes of various cell types.

### Single-cell RNA-seq elucidates stem cell identity in the antler tip

As the growth center, the tip tissue of the antler is crucial for rapid antler growth, ossification, and its periodic regeneration. We annotated two stem cells in the antler tip according to the marker genes mentioned in the recent literature, namely ASCs and ABPCs. Among them, ABPCs were identified as a special cell type distributed in both the mesenchymal and cartilage tissues of the antler growth center, and they were more prevalent in the mesenchymal tissue than in the cartilage tissue. To further interpret the gene expression characteristics and decipher the functions of ABPCs during antler tip elongation and ossification, we identified gene sets enriched in ABPCs and conducted functional enrichment analysis, providing strong evidence and valuable reference for understanding the dynamic gene expression patterns underlying antler elongation and ossification.

The genes that were highly expressed in ABPCs were primarily associated with extracellular matrix organization (GO:0030198) (*TNC*, *CDH11*, *COL5A1*, *COL1A2*, *ITGA11*, *DCN*, *DPT*, *POSTN*, *COL12A1*, *COL5A2*, *SEC24D*, and *ADAMTS2*), positive regulation of developmental growth (GO:0048639) (*PRICKLE2*, *NAV3*, *EFNA5*, and *TSHR*), positive regulation of organ growth (GO:0046622) (*NCAM1* and *CCN4*), ossification (GO:0001503) (*ESR1* and *KREMEN1*), cartilage development (GO:0051216) (*RUNX2*, *SATB2*, and *Prrx1*), and regulation of the Wnt signaling pathway (GO:0030111) (*CDH2* and *WNT5B*) (Supplementary Table S1), all of which are closely related to rapid antler elongation and ossification. As expected, this reflects the potential vital role of ABPCs in rapid antler elongation and ossification.

It is universally acknowledged that ASCs are capable of promoting antler elongation (30). To further investigate the characteristics and functions of ASCs in the antler tip, we identified genes that were significantly highly expressed in ASCs and performed functional enrichment analysis. The results revealed that genes enriched in ASCs exhibited strong proliferative activation. Among them, *IGF1*, one of the most effective growth factors that stimulates antler growth, was specifically expressed in ASCs (16, 33). Previous studies have demonstrated a significant positive correlation between *IGF1* and the antler growth rate (34), indicating that ASCs may play a positive regulatory role in promoting rapid antler growth. Interestingly, *RXFP2*, a gene related to horn growth (30), was also specifically expressed in ASCs. *RXFP2* plays a critical role in regulating sheep horn phenotypes, including size and shape (35, 36). It is specifically highly expressed in the horn and testicular tissue of bovids (37). *RXFP2* was also specifically found to be highly expressed in antlers (37). Therefore, the specific expression of *RXFP2* in ASCs indicates that ASCs play a crucial role in rapid antler growth. These findings also demonstrate that ASCs are closely related to rapid antler growth and the characteristics of their phenotypes. In addition, we also identified genes that are highly expressed in ASCs and involved in stem cell proliferation (GO:0072089), mesenchyme development (GO:0060485), and mesenchymal cell differentiation (GO:0048762), including *SFRP2*, *TGFBR3*, *FGFR2*, *EDNRA*, and *Bnc2*. Highly expressed genes in ASCs also exhibited an expression signature associated with bone development, such as ossification (GO:0001503), regulation of ossification (GO:0030278), and skeletal system morphogenesis (GO:0048705) (Supplementary Table S2), suggesting that ASCs may be vital for the formation of cartilage and bone in antlers and may have the potential to differentiate into chondrocytes and chondroblasts.

### Pseudotemporal analysis constructs the differentiation trajectories in the antler tip

**Figure 3:**
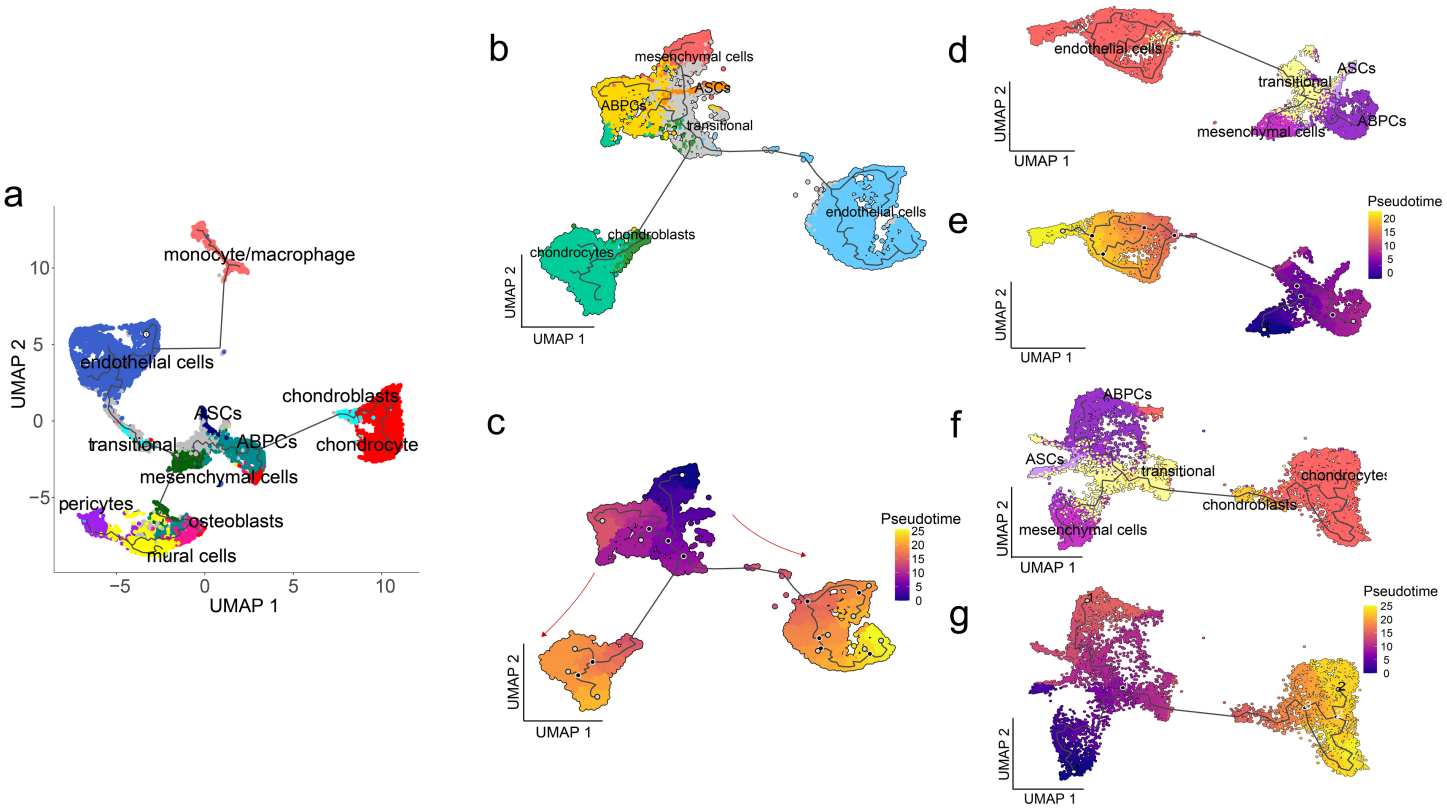
Reconstruction of the pseudotemporal trajectory in the antler tip. **(a)** Pseudotemporal trajectory analysis of 11 cells in the antler tip. **(b)** Pseudotemporal trajectory analysis of different cells in the antler tip, including mesenchymal cells, ASCs, ABPCs, transitional cells, endothelial cells, chondroblasts, and chondrocytes. Different cell types are marked by different colors. **(c)** Pseudotemporal trajectory analysis of different cells in the antler tip, and the cell color changes with pseudotemporal progression. **(d)** Pseudotemporal trajectory analysis of the transition from ABPCs to endothelial cells in the antler tip, with different cell types represented by different colors. **(e)** Pseudotemporal trajectory analysis of the transition from ABPCs to endothelial cells in the antler tip. The color of the cells changes with pseudotemporal progression. **(f)** Pseudotemporal trajectory analysis of the transition from ABPCs to chondrocytes and chondroblasts in the antler tip, with different cell types represented by different colors. **(g)** Pseudotemporal trajectory analysis of the transition from ABPCs to chondrocytes and chondroblasts in the antler tip. The color of the cells changes with pseudotemporal progression.

To investigate the relationships between various cell types, pseudotemporal analysis was performed on the 11 distinct cell types identified (Figure 3a). Among these cell types, we focused particularly on mesenchymal cells, ASCs, ABPCs, endothelial cells, transitional cells, chondrocytes, and chondroblasts. We observed an outward differentiation trajectory that initiated from ABPCs and mesenchymal cells during the differentiation process of the antler tip. Mining the pseudotemporal analysis results revealed three major branching paths (Figures 3b,c): one representing the transition from ABPCs and mesenchymal cells to endothelial cells (Figures 3d,e), another representing the transition from ABPCs and mesenchymal cells to chondrocytes and chondroblasts (Figures 3f,g), and a third involving the transition from ABPCs and mesenchymal cells to mural cells.

We further determined the gene sets that were differentially expressed along the differentiation trajectories. Compared to mesenchymal cells and ABPCs, we observed a large number of significantly upregulated genes in endothelial cells that were closely related to angiogenesis, such as *RASIP1*, *NRP1*, *RAMP2*, *Tbx1*, *EFNA1*, *Rhob*, and *SAT1*. In addition, many of the significantly upregulated genes in endothelial cells were enriched in several Gene Ontology (GO) terms, such as positive regulation of angiogenesis (GO:0045766), vasculogenesis (GO:0001570), regulation of angiogenesis (GO:0045765), and blood vessel development (GO:0001568) (Supplementary Figure S4). Some of the significantly upregulated genes in endothelial cells were also found to be involved in the GO term of endothelial cell proliferation (GO:0001938, GO:0001935) (Supplementary Figure S4), suggesting that these upregulated genes along the differentiation trajectory from ABPCs and mesenchymal cells to endothelial cells are vital for the formation of the vascular network in the antler tip. Notably, the expression of marker genes in mesenchymal cells gradually decreased along the trajectory pathway (Supplementary Figure S5), while the expression of marker genes in endothelial cells gradually increased (Supplementary Figure S5).

To explore the role of chondrocytes in the antler tip, we further identified upregulated genes in chondrocytes along the differentiation trajectory. Compared to ABPCs and mesenchymal cells, we observed that significantly upregulated genes in chondrocytes were enriched in multiple GO terms closely related to skeletal development, such as negative regulation of cell growth (GO: 0030308) and skeletal system development (GO: 0001501) (Supplementary Figure S6). The significantly upregulated genes were also correlated with cartilage development (GO:0051216, *SOX9*, *FBXW4*, *CNMD*, *ZBTB16, MGP*, *CFAP65*, and *Csgainact1*) and skeletal muscle cell differentiation (GO:0035914, *MEF2C*, *FOXN2*, *FOS*, *NR4A1*, *PLAGL1*, and *CITED2*) (Supplementary Figure S6). These upregulated genes associated with bone development may play a crucial role in endochondral ossification during rapid antler elongation. The expression of marker genes in chondroblasts and chondrocytes increased along the differentiation trajectory (Supplementary Figures S7, S8). In contrast, the expression of marker genes in ABPCs and mesenchymal cells decreased along the differentiation trajectory (Supplementary Figure S7).

### Ligand–receptor interaction relationship of the antler tip

**Figure 4:**
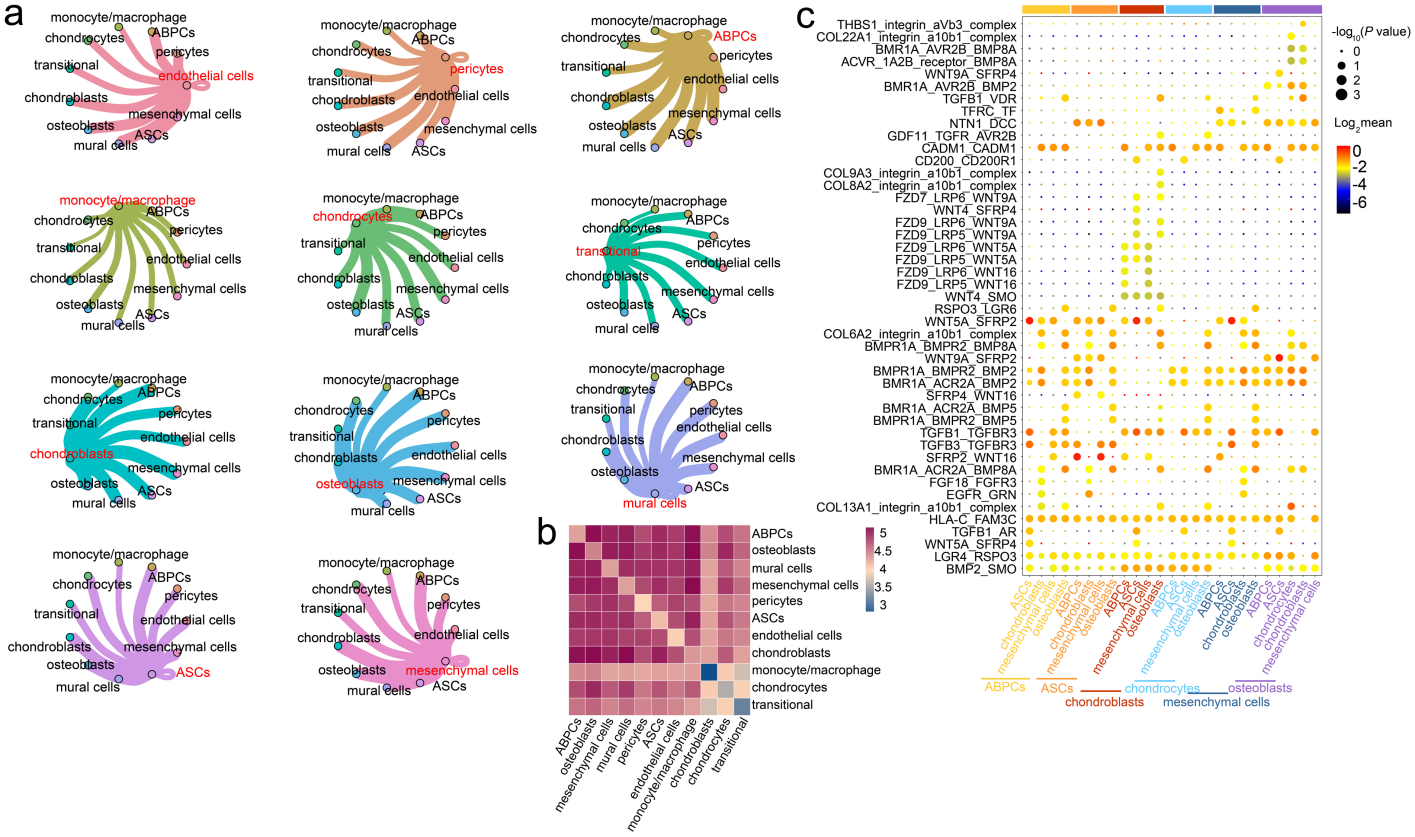
Prediction of intercellular ligand–receptor interactions in the antler tip. **(a)** Interaction network among diverse cells in the antler tip. Each color represents the interaction network between one cell type and the other ten cell types. The width of the connecting line represents the intensity of the interaction between two cell types. **(b)** Heatmaps of interactions among different cell types (*p* < = 0.05). **(c)** Dot plots showing ligand–receptor pairs among different cell types. Modules with different colors on the vertical axis represent different cell types.

The communication between various cell types provided significant insights into the possible molecular mechanisms of rapid antler growth and ossification. We therefore constructed a cell–cell interaction network among diverse cells and predicted the potential ligand–receptor pairs in the antler tip. The results indicated that three cell populations with co-expression relationships had strong intercellular communication with chondroblasts, namely ABPCs, mesenchymal cells, and ASCs (Figures 4a,b). Among these cell populations, ABPCs also exhibited strong interactions with other cell types, such as mural cells, osteoblasts, and transitional cells (Supplementary Figure S9). Mesenchymal cells and ASCs also exhibited interactions with osteoblasts, mural cells, and transitional cells (Supplementary Figure S9).

We then predicted potential ligand–receptor pairs on the single-cell transcriptomic landscape for understanding the ligand–receptor interaction among different cell types in the mesenchymal and cartilage tissues of the antler tip. The results revealed that some ligand–receptor pairs were significantly expressed only in certain cell types. For example, ASCs expressed high levels of *SFRP4*, whose receptors included the members of the Wnt family, such as *WNT4* and *WNT9A*. The WNT4/SFRP4 ligand–receptor interaction pair was highly expressed in chondroblasts and ASCs, while the WNT9A/SFRP4 ligand–receptor interaction pair was highly expressed in osteoblasts and ASCs (Figure 4c). Additionally, osteoblasts and chondroblasts expressed high levels of the integrin_a10b1_complex, whose receptors included members of the collagen family, such as *COL22A1*, *COL8A2*, and *COL9A3*. Other significant ligand–receptor interaction pairs were shared by the vast majority of cell types, such as the CADM1/CADM1, TGFB1/TGFBR3, LGR4/RSPO3, and HLA-C/FAM3C ligand–receptor interaction pairs (Figure 4c). These ligand–receptor interaction pairs, shared by the vast majority of cells, provide significant references for exploring the molecular basis of cell-to-cell communication in the antler tip.

## Discussion

In the present study, our primary goal in mapping the single-cell transcriptomic landscape was to reveal its cellular composition and differentiation trajectories. This provides a novel dimension to antler developmental biology, particularly regarding the rapid elongation and differentiation of the antler tip. Within our dataset, a total of 11 distinct cell types were annotated in the mesenchymal and cartilage tissues of the antler growth center, including stem cell-like state cells, multifunctional immune cells, proliferative cells, chondrocytes, chondroblasts, and transition cells. However, some other cell types may not have been detected due to unavoidable factors, such as a lack of dissociation or cell death (36). Pseudotemporal analysis of diverse cells revealed that the rapid growth and ossification of the antler primarily consists of three differentiation paths: First, the transition from ABPCs to endothelial cells; second, the transition from ABPCs to chondrocytes and chondroblasts; and third, the transition from ABPCs to mural cells. Various cells maintain the rapid elongation of the antler and promote the differentiation process from mesenchymal cells to chondrocytes by communicating with each other. Our study thus provides a novel insight and perspective into the cellular composition and the underlying molecular mechanisms of the rapid growth and ossification of antlers.

In this study, numerous endothelial cells were identified in the mesenchymal and cartilage tissues of the antler tip. A recent study showed that endothelial cells play a vital role in the formation of the vascular network during antler regeneration (15). Similarly, a study on axolotl limb also demonstrated that endothelial cells may contribute to its re-vascularization (37). Antler cartilage with abundant blood vessels, unlike other cartilage tissues, has unique biological characteristics of self-repair and regeneration (12). Notably, the blood vessels located in the growth center of the antler elongate at a very fast rate during periods of rapid antler growth (11, 38, 39). Therefore, the large number of endothelial cells identified in the growth center of the antler plays a significant role in the formation of the vascular network during the rapid elongation of the antler, which also contributes to the high metabolic demands associated with antler growth (39).

Rapid antler elongation primarily depends on cell proliferation and endochondral ossification in the antler growth center, enabling the antler to develop into a unique bone in the mammalian kingdom. Unlike the recently published research on the cellular composition of antler tip tissues (15, 16), we identified ASCs in antler tips. These are a type of stem cell-like cell. ASCs can self-renew and eventually differentiate into various cells due to their high proliferative and regenerative abilities (30, 40, 41), which are considered to contribute to the annual periodic regeneration and rapid growth of antlers (11). Additionally, recent research has identified ABPCs with strong regenerative capacity and defined them as a stem cell pool for antler growth (16). ABPCs can also differentiate into cartilage cells due to their strong self-renewal ability (16). Consistent with these findings, we observed that the highly expressed genes in ASCs and ABPCs are primarily involved in cell proliferation and bone development, underscoring the crucial role of ABPCs and ASCs in rapid antler elongation and ossification. On the other hand, previous studies have shown that mesenchymal stem cells can differentiate into endothelial cells *in vitro* due to their strong differentiation ability (42). We also found genes involved in maintaining pluripotency (MYBL2 and DNMT1) in mesenchymal cells (43). Based on the aforementioned results, mesenchymal cells may play a potentially important role in rapid antler elongation.

The results of pseudotemporal analysis showed that the rapid antler growth and ossification can occur via three main pathways. First, one path involves a differentiation process from stem cell-like state cells (ABPCs, ASCs, and mesenchymal cells) to chondroblasts and chondrocytes, which is consistent with endochondral ossification during rapid antler elongation. Second, another pathway involves a differentiation process from stem-cell-like state cells to mural cells, and this process has been experimentally confirmed (44). Third, a pathway involves a differentiation process from stem-cell-like state cells to endothelial cells. However, from the perspective of embryonic development, it is worth noting that both ABPCs and mesenchymal cells in antlers are derived from the cranial neural crest (45), making them ectodermal in origin, whereas endothelial cells originate from the mesoderm. Therefore, whether ABPCs and mesenchymal cells can differentiate into endothelial cells remains to be experimentally validated in the future.

In general, complex biological processes cannot be tightly and finely regulated by a single cell type. The coordination between diverse cell types is essential for elucidating the biological function of an organism. The communication between different cells in the antler tip also clarifies the process of rapid antler growth and ossification. ABPCs, ASCs, and mesenchymal cells exhibit strong interaction with one another. This is also confirmed by co-expression modules. In addition, they have strong interactions with mural cells and osteoblasts. We also predicted the potential ligand–receptor interaction pairs in the antler tip. The WNT4/SFRP4 and WNT9A/SFRP4 ligand–receptor interaction pairs were highly expressed in chondroblasts and ASCs and osteoblasts and ASCs, respectively. Previous studies have shown that the Wnt signaling pathway is crucial for promoting cell proliferation and differentiation and maintaining the activity of stem cells. It is also involved in the occurrence and development of various tumors (46–48). As a member of the largest family of Wnt inhibitors, *SFRP4* competitively binds to Wnt factors (such as *Wnt3A*, *Wnt8A*, *Wnt9A*, and *Wnt10A*) to inhibit the Wnt signaling pathway and influence endochondral ossification (49), suggesting that the WNT4/SFRP4 and WNT9A/SFRP4 ligand–receptor interaction pairs may play a critical role in antler endochondral ossification We also found that the CADM1/CADM1 and TGFB1/TGFBR3 ligand–receptor interaction pairs were shared across the majority of cell types. Among these, CADM1 could be used as a tumor suppressor gene to promote apoptosis and inhibit the proliferation of cancer cells in multiple tumors (50, 51). *TGFB1* regulates the growth and differentiation of multiple cell types. Therefore, the CADM1/CADM1 and TGFB1/TGFBR3 ligand–receptor interaction pairs may regulate the rapid elongation of the antler and finely modulate its resistance to carcinogenesis. In summary, although these ligand–receptor interactions require to be further verified, we believe that the present study provides important insights and serves as a valuable reference for understanding the biological processes of rapid elongation and endochondral ossification in antlers.

## Data Availability

The raw sequencing data generated in the present study have been deposited in the China National Center for Bioinformation (CNCB) (GSA: CRA011335). All data included in this study are available upon request by contact with the corresponding author.

## Supplementary material

SUPPLEMENTARY TABLE S1 GO enrichment analysis of highly expressed genes in ABPCs.

SUPPLEMENTARY TABLE S2 GO enrichment analysis of highly expressed genes in ASCs.
