# Supplementary material for: Single-cell transcriptome analysis reveals elongation and ossification characteristics of antlers
Source: Front Vet Sci. 2025 Nov 26;12:1658210. doi: 10.3389/fvets.2025.1658210 (PMC12690211; doi:10.3389/fvets.2025.1658210)
Supplement: Supplementary file 1 [file Table_1.DOC]

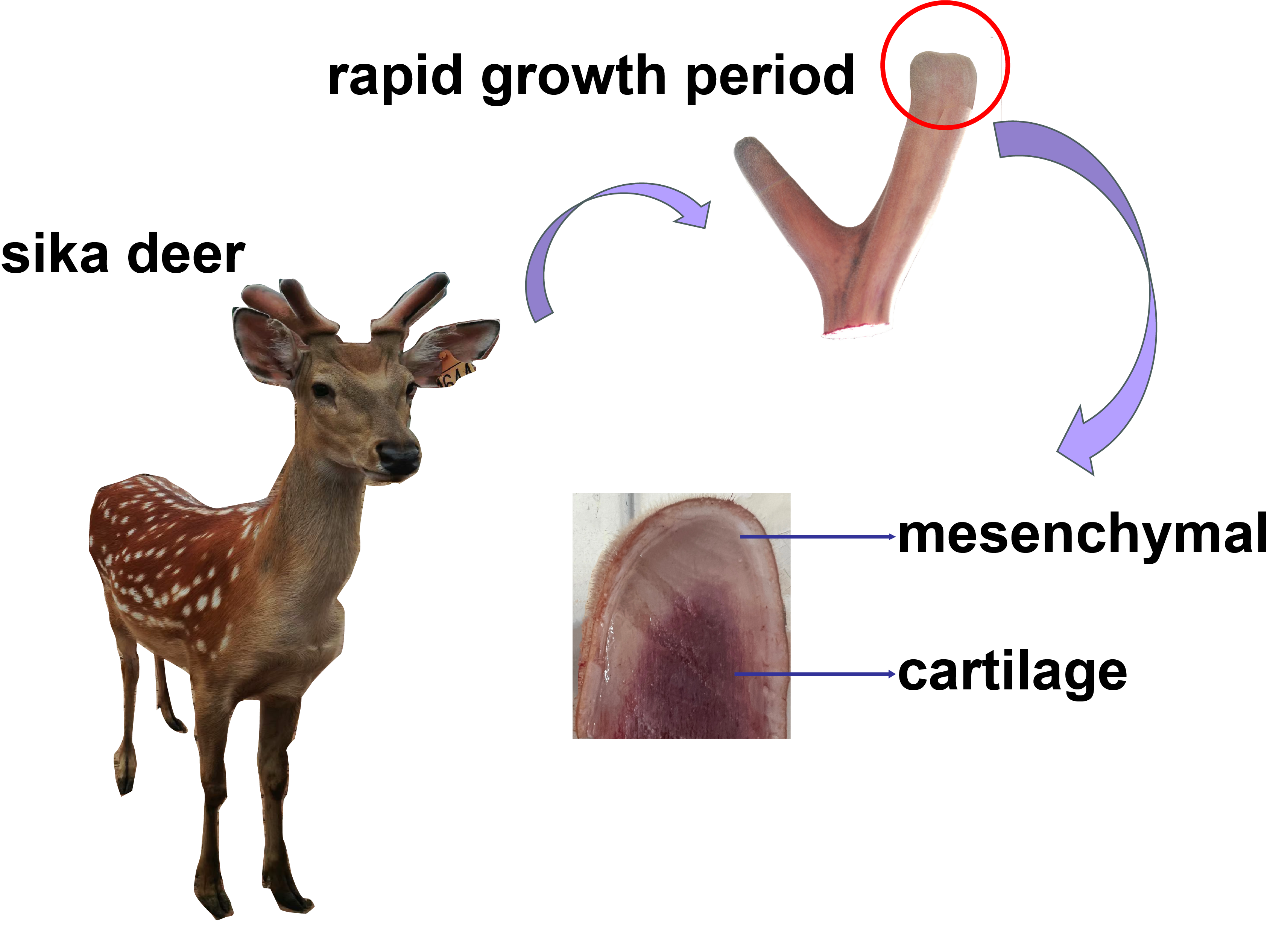


**Figure S1. Schematic of samples collected.**


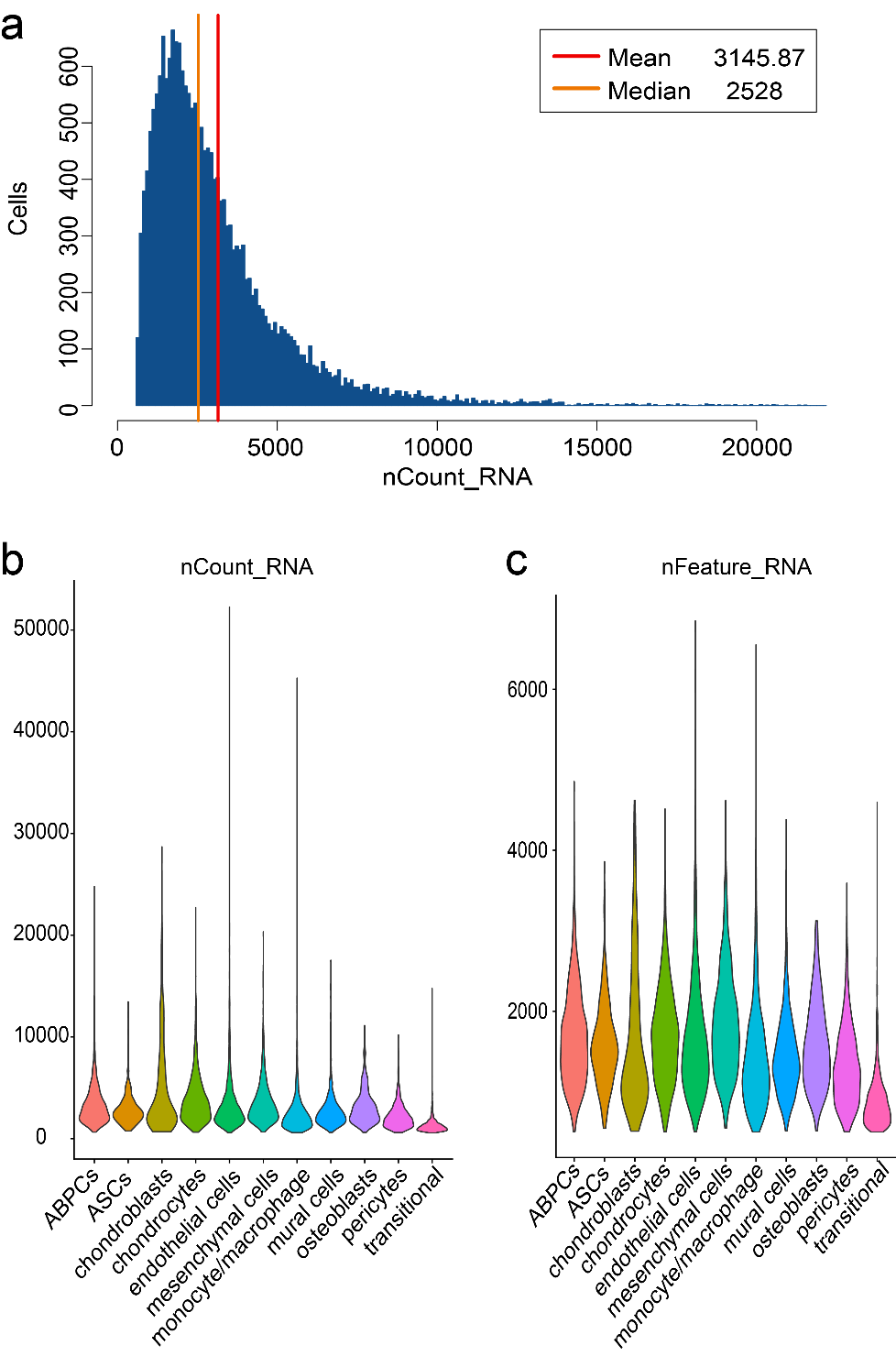


**Figure S2. Overview of scRNA-seq in the antler tip.**

(a) Histogram show the mean and median reads in each cell. (b) Violin plot show the count number of UMIs in each cell type. (c) Violin plot show the feature in each cell type.


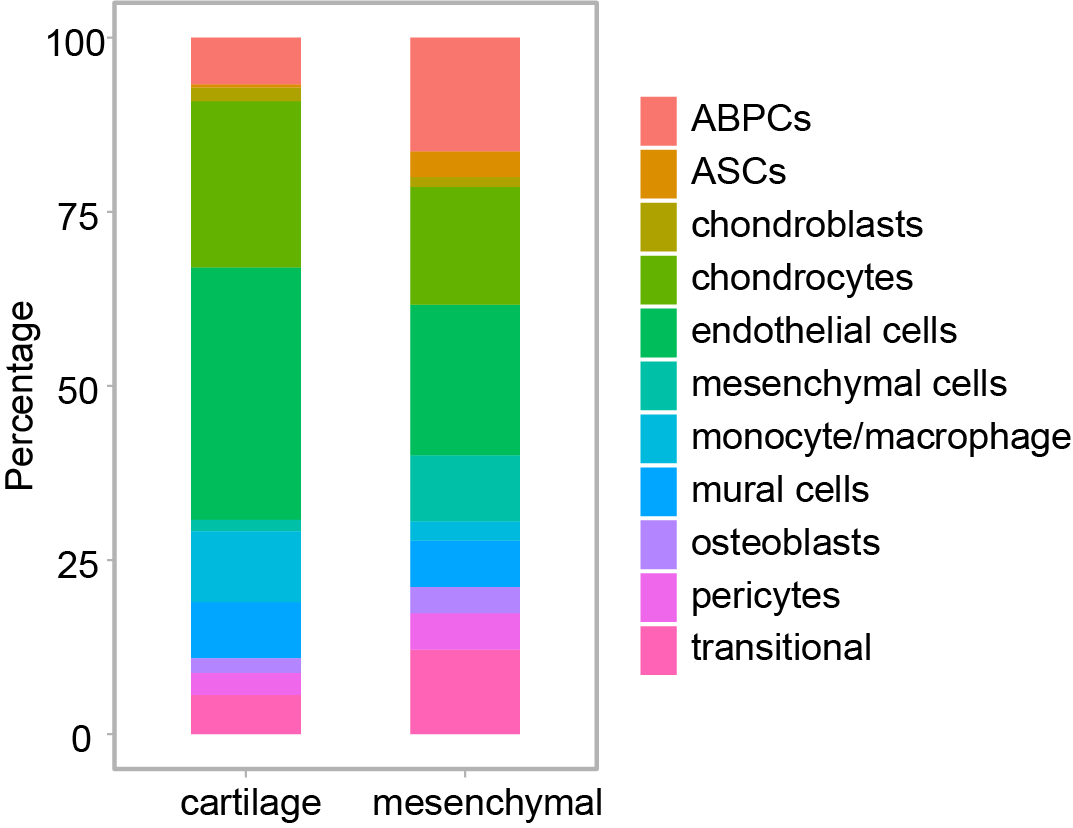


**Figure S3. The proportion of different cell types in the mesenchymal and cartilage tissues of antler.**

Different colors represent different cell types.


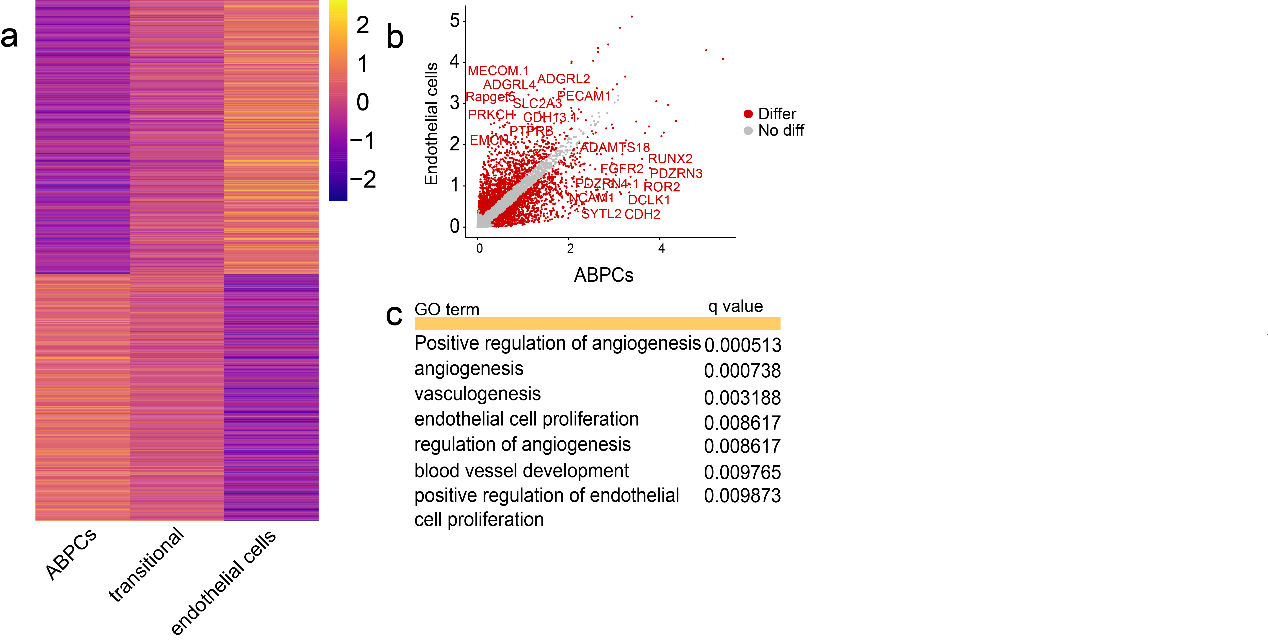


**Figure S4.** **Dynamic gene expression in the differentiation process from ABPCs into endothelial cells.**

(a) Heatmap of significantly differentially expressed genes during the differentiation from ABPCs to endothelial cells. (b) Scatter plot of gene expression between ABPCs and endothelial cells. Each dot represents one gene. The red dot represents genes that are significantly differentially expressed between two cell types, and the gray dot represents genes that are not significantly differentially expressed between ABPCs and endothelial cells. The labeled gene is the top gene with the most significant differences. (c) GO enrichment analysis of significantly up-regulated genes in endothelial cells compared with ABPCs.


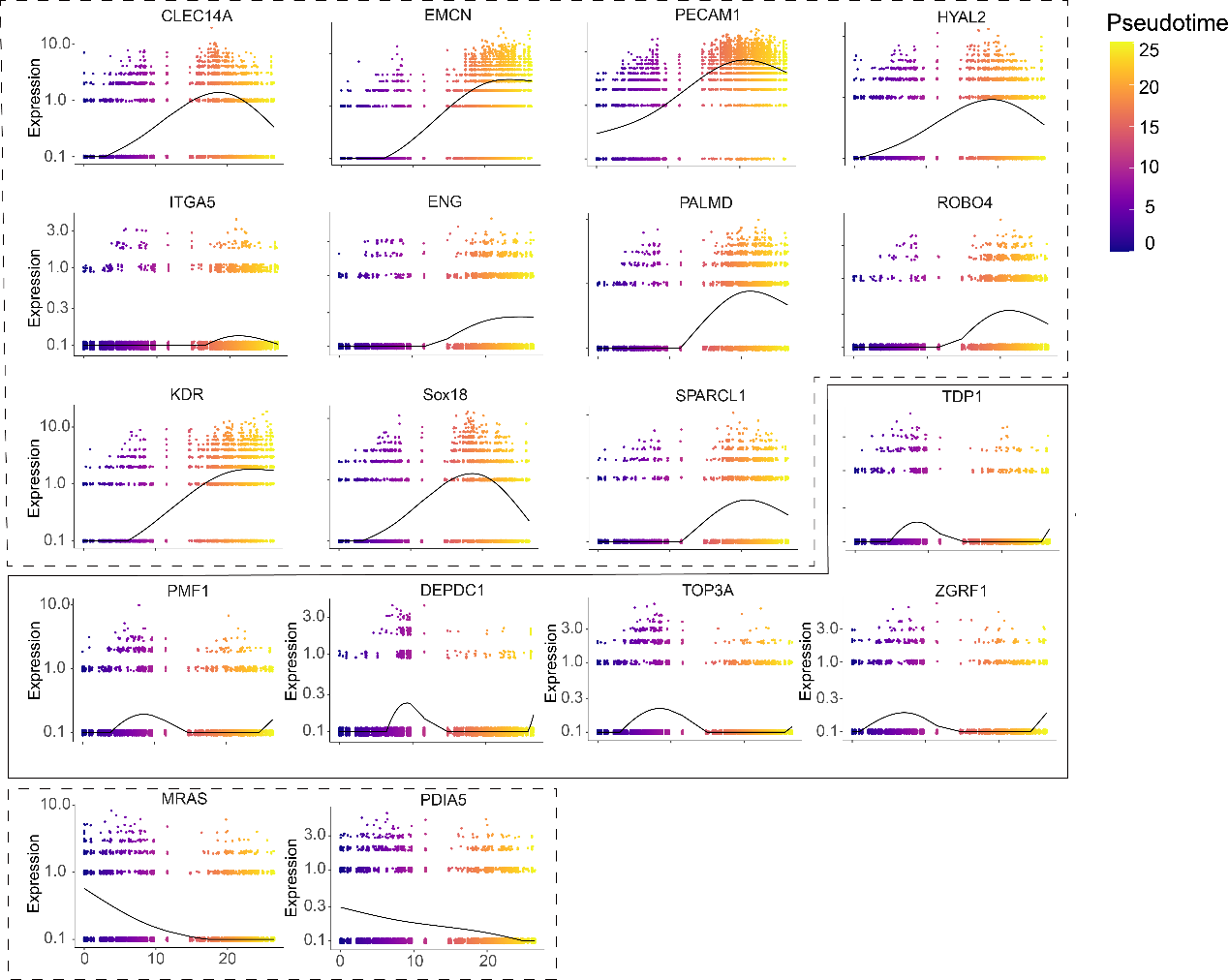


**Figure S5.** **The expression of marker genes in ABPCs, mesenchymal cells, and endothelial cells along pseudotime.**

The first dashed line represents the marker genes in endothelial cells, the second solid line shows the marker genes in mesenchymal cells, and the third dashed line area shows the marker genes in ABPCs. Cells were color-coded with different differentiation states.


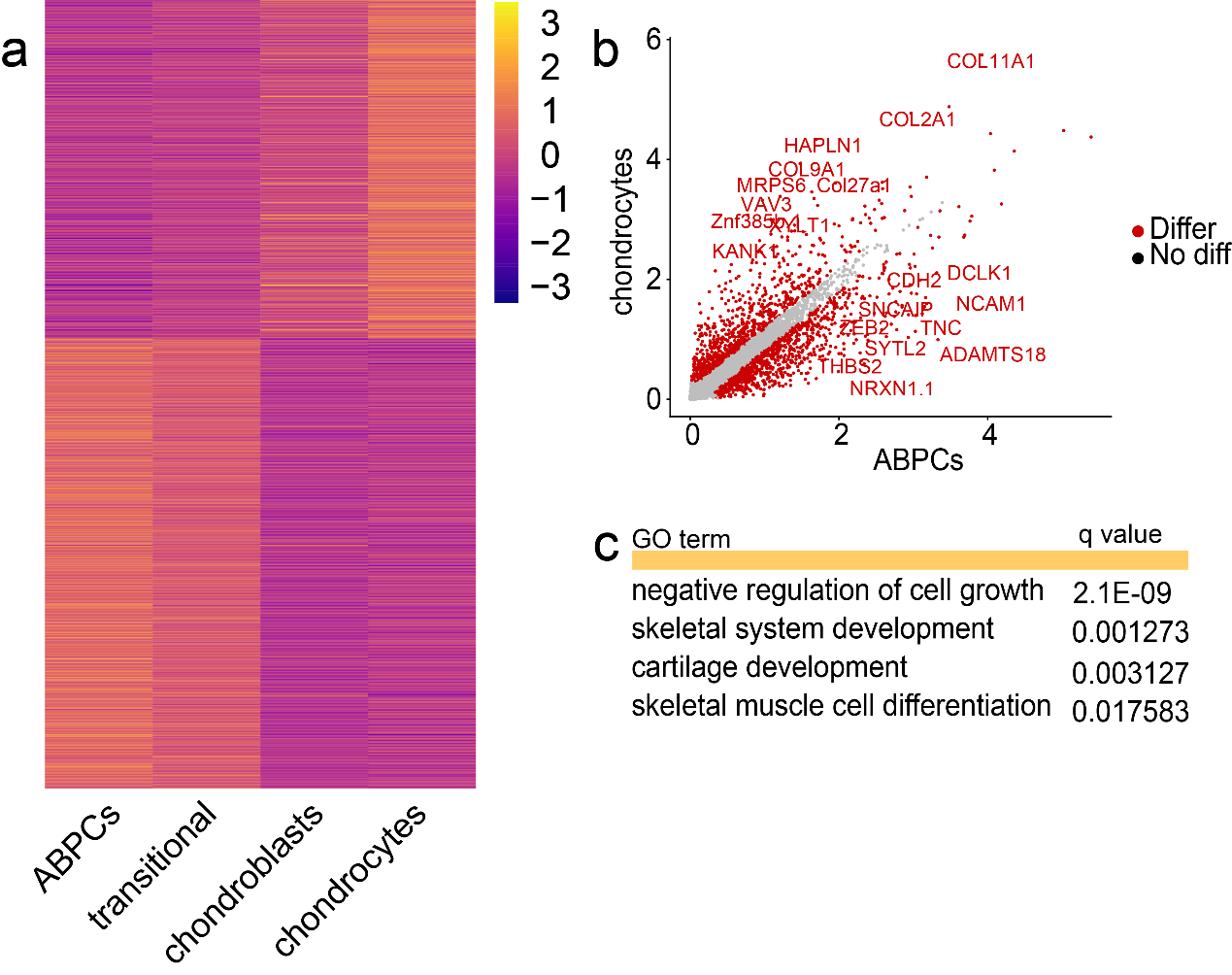


**Figure S6.** **Dynamic gene expression in the differentiation process from ABPCs into chondrocytes.**

(a) Heatmap of significantly differentially expressed genes during the differentiation from ABPCs to chondrocytes. (b) Scatter plot of gene expression between ABPCs and chondrocytes. Each dot represents one gene. The red dot represents genes that are significantly differentially expressed between two cell types, and the gray dot represents genes that are not significantly differentially expressed between ABPCs and chondrocytes. The labeled gene is the top gene with the most significant differences. (c) GO enrichment analysis of significantly up-regulated genes in chondrocytes compared with ABPCs.


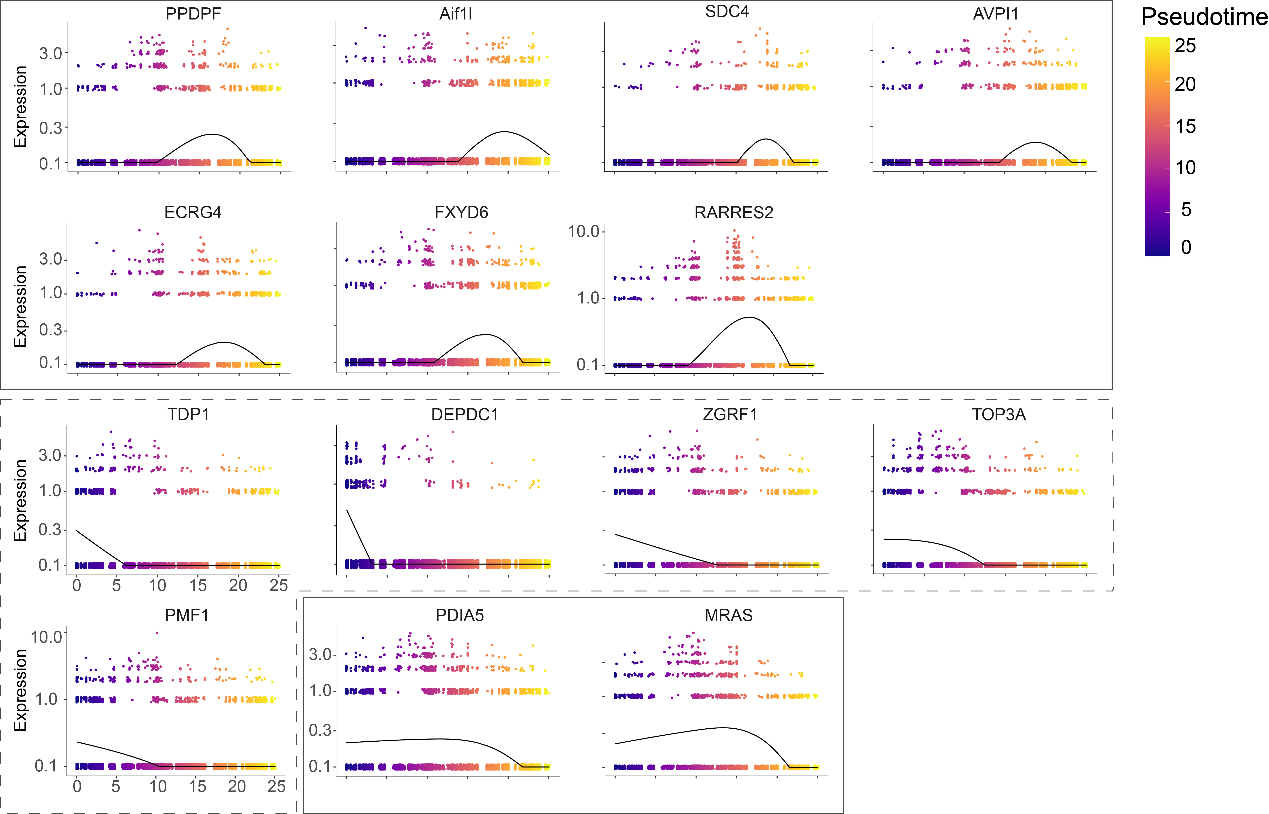


**Figure S7. The expression of marker genes in ABPCs, mesenchymal cells, and chondroblasts over pseudotime.**

The first dashed line shows the marker genes in chondroblasts, the second solid line represents the marker genes in mesenchymal cells, and the third dashed line area shows the marker genes in ABPCs. Cells were color-coded with different differentiation states.


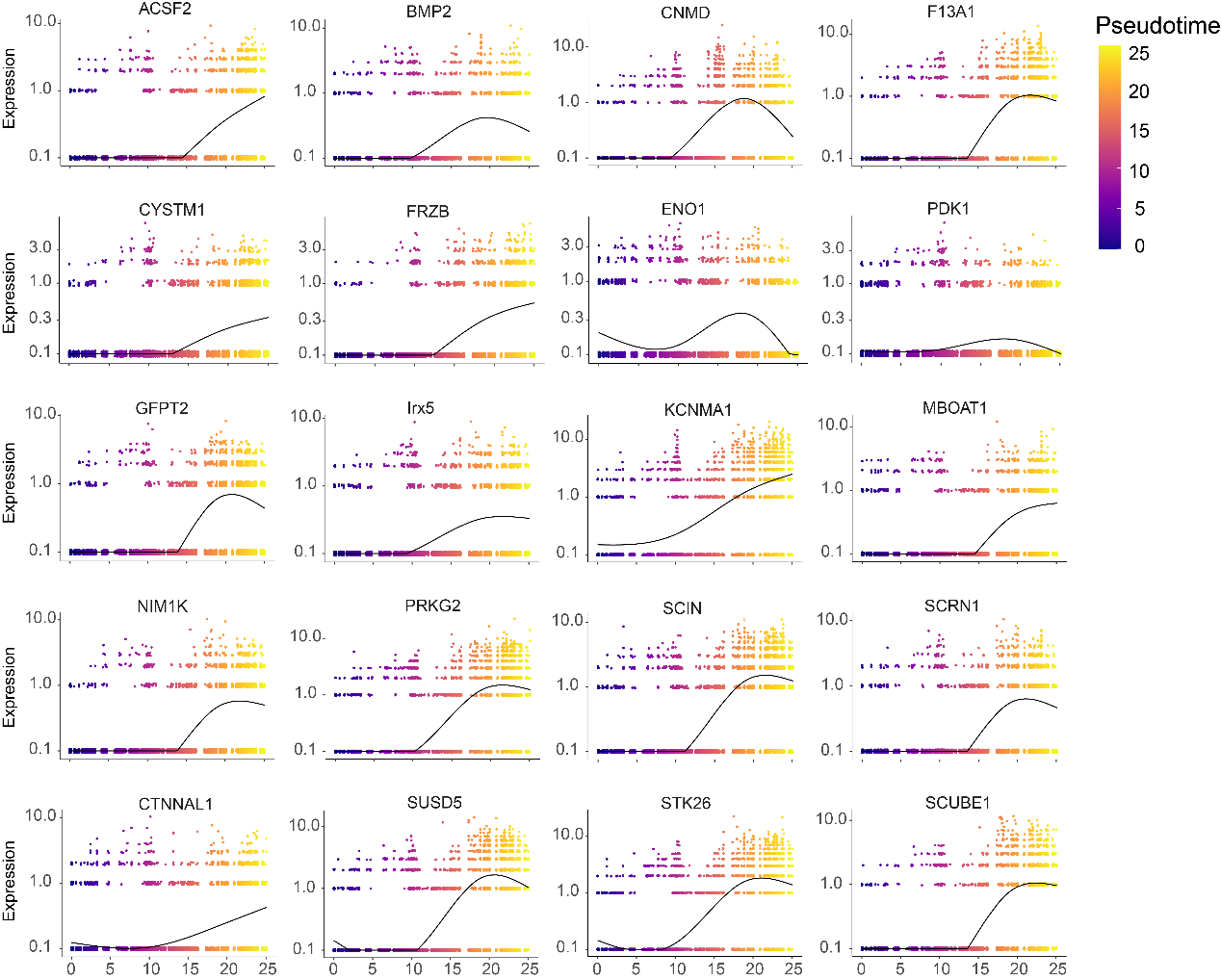


**Figure S8. The expression of marker genes in chondrocytes along pseudotime.**

Cells were color-coded with different differentiation states.


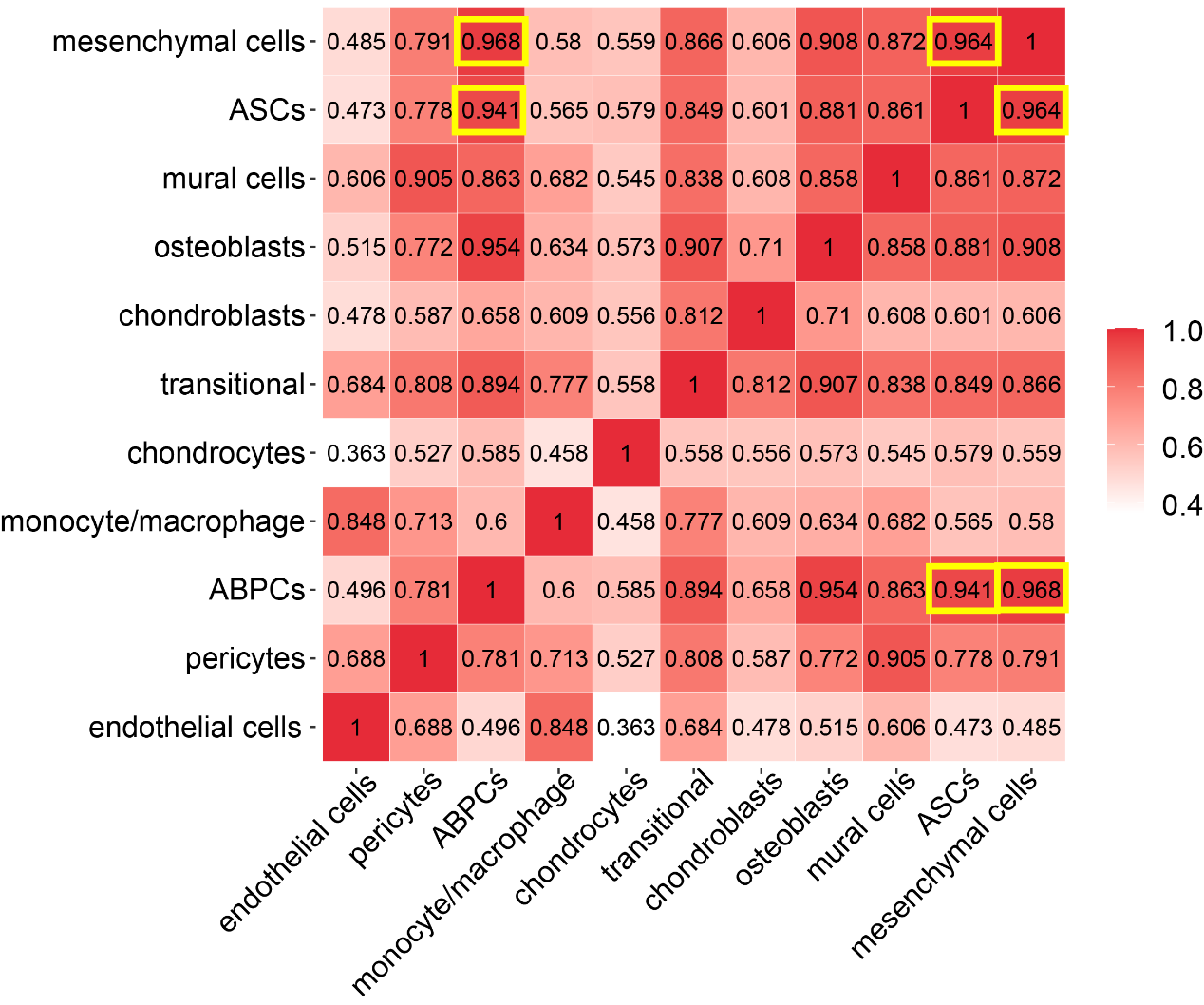


**Figure S9.** **Correlation coefficient between different cell types.**
